# Efficacy of a modified transvaginal ultrasound-guided fresh embryo transfer procedure

**DOI:** 10.5935/1518-0557.20210048

**Published:** 2022

**Authors:** Raul Nakano, Moacir R.M. Radaelli, Litsuko Shimabukuro Fujihara, Flávio Yoshinaga, Enzo Nakano, Carlos Gilberto Almodin

**Affiliations:** 1 Clínica de Reprodução Humana FERTICLIN, São Paulo, SP, Brazil; 2 Urology Department, Medical School, Faculdade Ingá, Maringá, PR, Brazil; 3 Materbaby - Reprodução Humana e Genética, Maringá, PR, Brazil

**Keywords:** transvaginal ultrasound, transabdominal ultrasound, fresh embryo transfer, pregnancy rates

## Abstract

**Objective:**

To present a modified transvaginal ultrasound (TVUS) guided embryo transfer (ET) procedure and analyze its efficacy in comparison with conventional transabdominal ultrasound (TAUS) guided ET in an unselected population of Brazilian women.

**Methods:**

This retrospective observational case-control study involved 447 fresh ET cycles, 221 guided by TVUS (Group 1), conducted between June 2016 and February 2019, and 226 by TAUS (Group 2), conducted between July 2012 and December 2015. Pregnancy rate was the main endpoint. Groups were compared using the Z test at a level of significance of 95% (*p*≤0.05).

**Results:**

Patient age ranged from 21 and 48 years; mean age was 37.7 years in Group 1 and 38 years in Group 2. Overall, patients that underwent TVUS-guided fresh ET demonstrated significantly higher pregnancy rates than their counterparts that underwent TAUS-guided fresh ET (*p*=0.0107). TVUS-guided fresh ET also yielded significantly higher pregnancy rates in the subgroups of women aged 36-39 years (*p*=0.0037) and ≥ 40 years (*p*=0.0025). However, no significant pregnancy rate difference was observed in women aged ≤ 35 years (*p*=0.0905).

**Conclusions:**

The results suggested that TVUS-guided fresh ET was at least as effective as TAUS-guided fresh ET in the studied sample. Pending further prospective studies to better ascertain the effect of TVUS-guided ET, the technique presented deserves consideration since it can offer better visualization, more comfort to patients, and requires only one operator, without negatively affecting pregnancy results.

## INTRODUCTION

The last step of an in vitro fertilization (IVF) cycle is the delivery of good quality embryos within the endometrial cavity. Compared to other IVF procedures, embryo transfer (ET) has hardly changed since it was first described by Edwards *et al*. (1984); consensus concerning the best ET protocol is yet to be reached (Mains & Van Voorhis, 2010; Toth *et al*., 2017).

Historically, the ET procedure involves inserting a catheter through the cervix and unloading the embryos somewhere within the uterine cavity. Also known as clinical touch ET, this blind procedure has progressively been replaced by ultrasound-guided ET, which has been shown by several meta-analysis conducted over the years to result in improved clinical pregnancy and implantation rates (Buckett, 2003; Sallam & Sadek, 2003; Abou-Setta *et al*., 2007; Brown *et al*., 2016). The exact mechanism by which ultrasound-guided ET is considered superior to clinical touch ET is still unclear. Among the reasons reported are the visualization of the tip of the catheter, which enables embryo deposition in the correct position within the uterus, the reduction of endometrial trauma, and the standardization of the procedure among physicians (Sallam & Sadek, 2003; Abou-Setta *et al*., 2007).

Based on a series of high quality randomized clinical trials (RCTs), a recent ET guideline by the American Society for Reproductive Medicine recommended its members should use two-dimensional transabdominal ultrasound (TAUS) during ET (Practice Committee of the American Society for Reproductive Medicine, 2017). A recent survey conducted with ASRM members demonstrated that the main reasons for the adoption of US guidance during ET were physicians' belief in improved success rates, and increased physician and patient reassurance (Toth *et al*., 2017).

Despite the apparent benefits provided by TAUS during ET, the procedure also presents some important drawbacks. Firstly, TAUS-guided ET requires an assistant to operate the transducer while a physician performs the ET procedure. Moreover, the patient needs to fill her urinary bladder to enable visualization of the uterine canal, which may cause discomfort and sometimes uterine cramping during ET, which in turn can negatively impact implantation rates (Fanchin *et al*., 1998; Lesny *et al*., 1998; Karavani *et al*., 2017). As a result, recent studies have turned their attention to transvaginal ultrasound (TVUS) as an alternative to TAUS-guided ET.

TVUS is the main technique used in all other gynecological investigations, in the assessment of follicle growth, and in oocyte retrieval (Larue *et al*., 2017a). Because of their high frequency and close proximity to the target area, TVUS transducers can provide better resolution of the uterocervical angle and improved overall image quality (Porat *et al*., 2010). Two previous RCTs comparing TVUS and TAUS guidance during ET reported similar results in terms of clinical pregnancy and implantation rates (Porat *et al*., 2010; Bodri *et al*., 2011). A recent systematic review found no differences in clinical and ongoing pregnancy rates and live birth rates between the two US techniques, and concluded that none was clearly better than the other in ET procedures (Cozzolino *et al*., 2018). In contrast, a recent large retrospective study indicated significantly higher pregnancy rates for patients that underwent TVUS-guided ET versus TAUS-guided ET (Larue *et al*., 2017a), while another recent RCT demonstrated that TVUS resulted in better visualization of the site of ET with patients reporting less pain and discomfort than with TAUS (Karavani *et al*., 2017).

Despite the potential benefits to both physicians and patients, TVUS-guided ET has not been widely adopted, probably on account of the difficulty in manipulating the ET catheter and the vaginal transducer simultaneously in a very constricted area (Larue *et al*., 2017a). Alternative approaches that may assist physicians to feel more comfortable during TVUS-guided ET should be explored.

Therefore, the objective of this study was to present a modified TVUS technique, and analyze its efficacy in comparison with conventional TAUS-guided fresh embryo transfer in an unselected population of Brazilian women.

## MATERIAIS AND METHODS

This retrospective observational case-control study was conducted to assess the efficacy of a modified technique employed in TVUS-guided ET, and to compare it to conventional TAUS-guided ET in an unselected population of Brazilian women who underwent IVF treatment at a private fertility clinic in the city of São Paulo, Brazil. The study was conducted in accordance with the ethical standards set out in Resolution 466/2012 of the Brazilian National Health Council, the 1964 Helsinki Declaration and its later amendments, and the recommendations set by the Strengthening the Reporting of Observational Studies in Epidemiology (STROBE) guidelines (von Elm *et al*., 2014). The local Institutional Review Board approved the study; since it is a retrospective study, informed consent was not required.

### Study population

The analysis was based on data extracted from the medical records of all women submitted to a modified TVUS-guided ET procedure between June 2016 and February 2019. Data from the records of a matching number of unselected women who underwent conventional TAUS-guided fresh ET procedures between July 2012 and December 2015 were used for comparison. Patients who underwent freeze all, made use of pre-embryos or devitrified oocytes, and egg-donation recipients were excluded from the sample. All procedures were conducted under the supervision of the same physician.

### Ovarian stimulation

All patients had their ovaries stimulated following the same protocol, always performed by the same experienced physician. From Day 2 of the menstrual cycle, the patients received a daily dose of urinary FSH (Fostimon, UCB) 300 IU, followed by 0.25 mg/day of GnRH antagonist (Orgalutran, MSD) from the moment follicles reached ≥ 13 mm in diameter. Triggering was started when at least two leading follicles measuring ≥ 20 mm were observed with the administration of hCG 10,000 IU (Choriomon, UCB).

Thirty-four hours after triggering, the patients underwent follicular aspiration under sedation with 50 mg of propofol 1% (Diprivan^®^, AstraZeneca, Brazil) under TVUS guidance. The retrieved oocytes were immediately sent to the embryology laboratory where they were placed in an incubator with 6% CO_2_ at 37ºC, and one to two hours later into hyaluronidase (IngaMed, Maringá, Brazil) for the removal of the surrounding cumulus and corona cells. The same experienced embryologist counted and categorized the harvested oocytes according to the criteria established by the Society for Assisted Reproductive Technology (Racowsky *et al*., 2010).

### ICSI

MII oocytes underwent intracytoplasmic sperm injection (ICSI) using polyvinylpyrrolidone (PVP - IngaMed, Maringá, Brazil) four hours after follicular aspiration. Embryo culture was conducted in a 50-µl drop of culture medium (GV-Blast^®^ - IngaMed, Maringá, Brazil) under oil. Fertilization was assessed 18-20 h after ICSI, and the embryos were kept in culture until day three post-fertilization (Almodin *et al*., 2010). Embryos were categorized as Grade I (6-8 cells, showing similarly-shaped blastomeres and no fragmentation) or Grade II (6-8 cells and similarly-shaped blastomeres with ≤20% fragmentation) according to Alikani *et al*. (1999). Only one or two embryos were selected for fresh ET, while the remainder were vitrified and stored in a nitrogen tank.

Hormonal support was started on the day after oocyte collection, either with intramuscular progesterone injections 50 mg/day (West-Ward Pharmaceuticals, NJ, USA) or 800 mg/day of micronized vaginal progesterone (Utrogestan^®^ - Farmoquimica, São Paulo, Brazil).

### Fresh embryo transfer

Fresh ET procedures were performed using a Sydney IVF catheter (Guardia(tm), Cook^®^ Medical, Australia). This catheter has a stiffer outer sheath, which is used to guide the softer inner catheter that carries the embryos inside the endometrial cavity.

The patient was placed in a gynecological position and a disposable speculum lubricated with paraffin oil was inserted into the vagina to expose the cervix. The external orifice was cleaned with phosphate-buffered saline medium (Dulbecco's PBS solution; Irvine Scientific) and a cotton swab, and cervical mucus removed with a 1 ml insulin syringe. Then, the outer rigid part of the catheter was carefully passed through the cervical canal into the uterine cavity up to approximately 20-25 mm from the fundus under US guidance conducted using a Toshiba Nemio XG SSA-580A ultrasound machine (Toshiba, USA).

Meanwhile, as soon as the laboratory received the signal, the flexible inner catheter was prepared for fresh ET within a laminar flow under sterile conditions at 37ºC. First, a 1-ml syringe was filled with warmed, aerated culture medium and attached to the catheter. The plunger was pressed to wash the catheter and remove manufacturing residues. With the catheter completely filled with culture medium, final assembly was accomplished as follows: first a small air bubble, followed by the medium containing the embryos, followed by another small air bubble, with the final volume not exceeding 20 µl. When ready for transfer, the catheter with the embryos was delivered to the physician by a nurse through the pass-through.

After the completion of fresh ET, the catheter was gently removed and immediately returned to the laboratory to ensure that no embryos had been retained inside, and to assess the presence of mucus and blood. In case of returned embryos, the catheter was immediately refilled and reintroduced in the patient. The patients remained resting in bed for approximately 10 minutes at the completion of the procedure.

### TVUS-guided fresh ET

**Figure 1:**
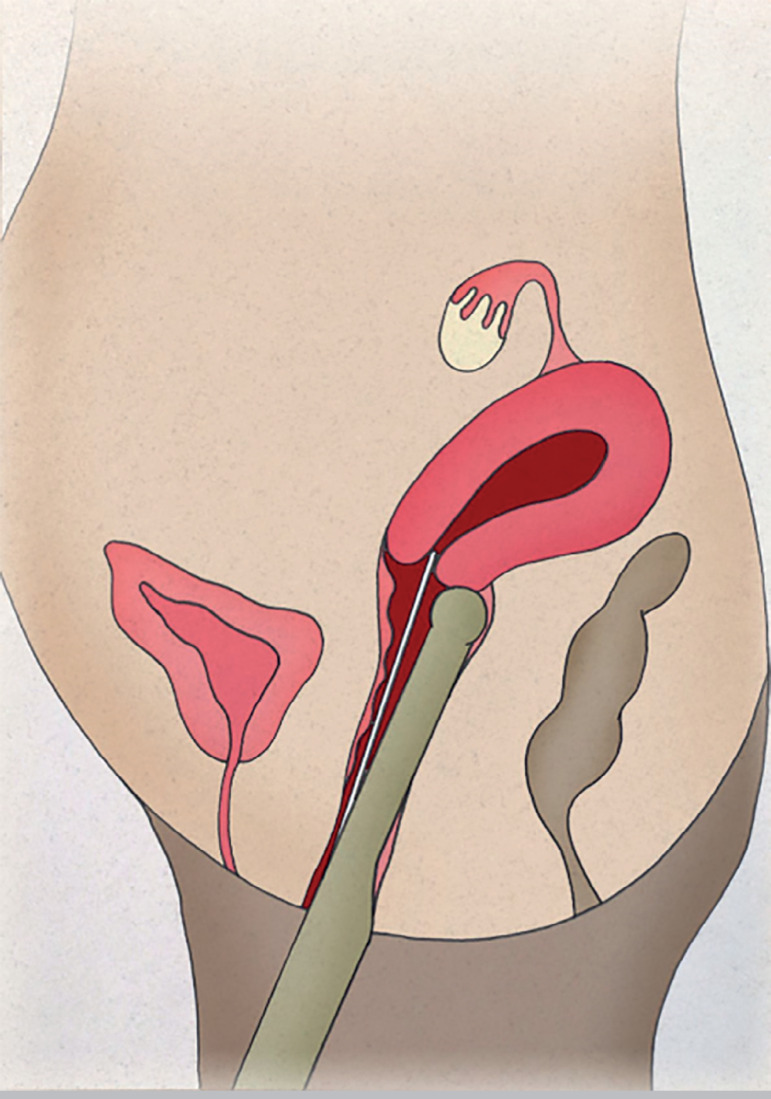
Retroverted uterus. Positioning of the head of the transducer on the vaginal fundus on the lower part of the cervix.

**Figure 2:**
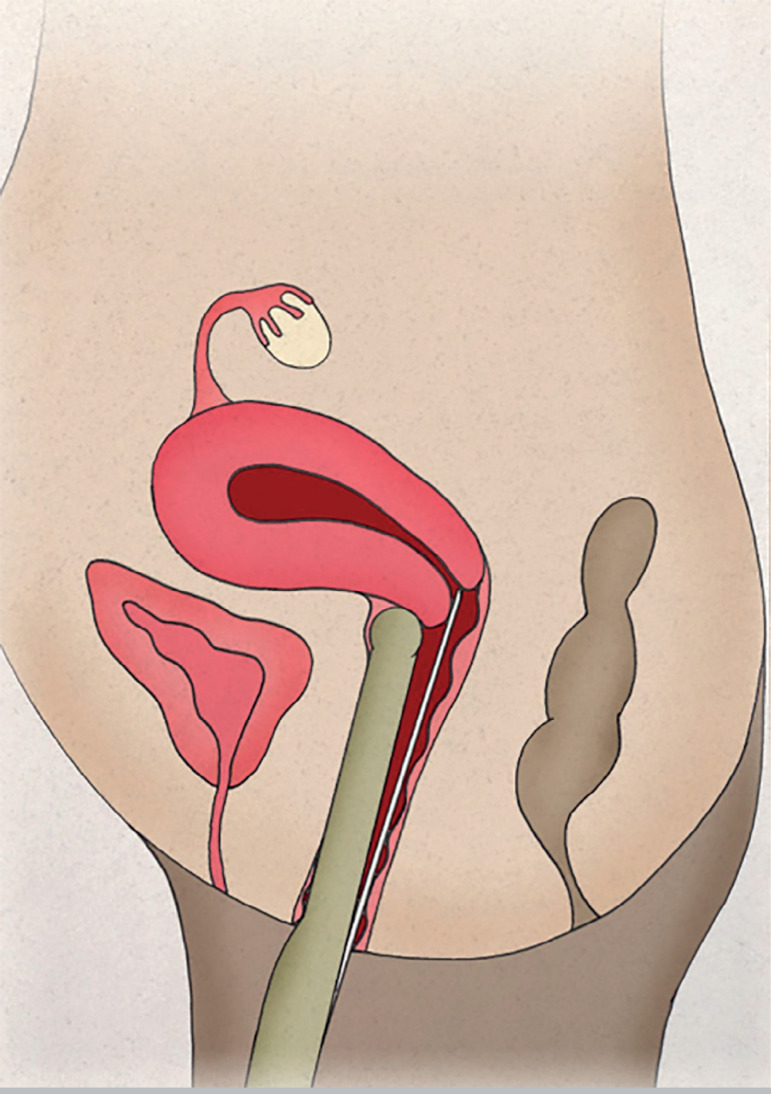
Retroverted uterus. Positioning of the head of the transducer on the vaginal fundus on the lower part of the cervix.

The patients were instructed to empty the bladder before entering the surgery room. Only one experienced professional was required to monitor the entire intrauterine path taken by the catheter containing the embryos. The vaginal transducer (PVF-621VT, Toshiba, USA) was covered with a sterile condom containing ultrasound transmission gel. The TVUS transducer maintained a direct sagittal angle of the cervix and uterus to permit the visualization of the entire endometrial cavity, from the internal orifice to the uterine fundus. The head of the transducer was positioned on the vaginal fundus either in the lower part of the cervix in cases of retroverted uteruses (Fig. 1), or on the upper part of the cervix in cases of anteverted uteruses (Fig. 2), allowing the catheter to pass freely through the cervix. Under TVUS guidance, the outer rigid part of the catheter was placed in the cervical canal, until the catheter tip was properly positioned. With the back of his left hand facing the patient, the physician held the TVUS transducer and the outer catheter between his thumb and index fingers, keeping it secure and in position. Then, the physician took the flexible inner catheter previously prepared with the embryos with his right hand and backloaded it into the rigid outer catheter.

**Figure 3:**
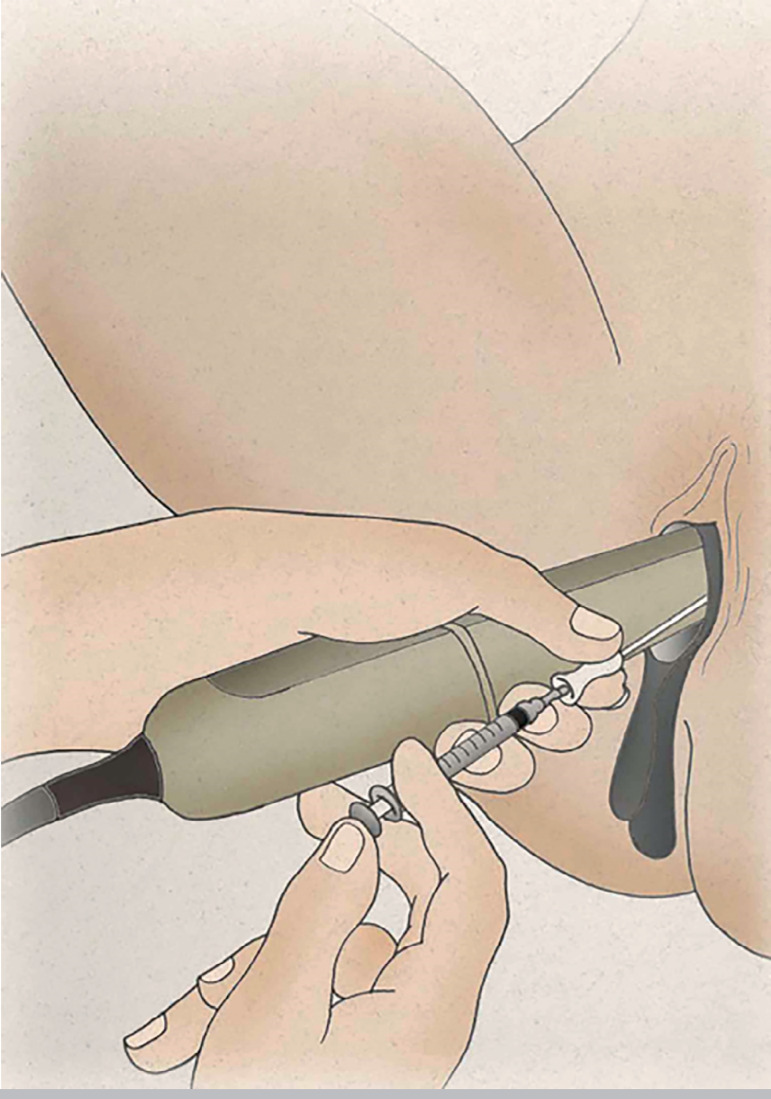
Culture medium with the embryos being slowly deposited in the uterus by pressing the syringe plunger under TVUS guidance.

While holding the TVUS transducer and the outer catheter with his left hand, the tip of the inner catheter was very gently advanced or withdrawn with the right hand to overcome any resistance, always being careful to avoid touching the fundus. When the tip of the catheter was 1.5-2 cm from the uterine fundus (Tiras *et al*., 2010), the culture medium with the embryos was slowly deposited by pressing the syringe plunger with the same hand; the plunger was kept pressed until the catheter was totally removed (Fig. 3). A hyperechogenic image of the two small air bubbles, indicating the place where the pre-embryos were retained in the fundus, was observed on the screen.

### TAUS-guided fresh ET

The patients were instructed to fill the bladder one hour prior to the procedure. The time to start fresh ET was individualized, since appropriate visualization could only be obtained when the bladder was completely distended. TAUS-guided fresh ET was conducted with a convex abdominal transducer (PVM-625AT, Toshiba, USA) operated by an experienced assistant. Under TAUS guidance, maintaining a longitudinal section of the uterus to permit visualization of the catheter as it passed through the cervix, the physician introduced the outer rigid catheter into position in the uterine cavity. Then, the physician received the inner catheter from a nurse and maneuvered it to the right position, delivering the embryos in the uterine cavity 1.5-2 cm from the uterine fundus (Tiras *et al*., 2010), as previously described. A hyperechogenic image of the two small air bubbles, indicating the position of the embryos in the uterine cavity was observed on the screen.

### Endpoint

The main endpoint was pregnancy, confirmed when β-hCG levels 14 days after fresh ET were greater than 100 IU/ml.

### Statistical analysis

Data collected were analyzed with the aid of statistical package *Statistica* 13.2 single user (TIBCO Statistica^®^ - Palo Alto, CA, USA). Overall pregnancy rates and the rates for the subgroups of women aged ≤ 35 years, between 36 and 39 years, and ≥ 40 years obtained from patients in Group 1 (TVUS) and Group 2 (TAUS) were compared using the Z test, with the level of statistical significance set at *p*<0.05.

## RESULTS

A total of 447 fresh ET cycles, 221 guided by TVUS (Group 1) and 226 by TAUS (Group 2) were performed. Patient age ranged from 21 to 48 years; mean ages in Groups 1 and 2 were 37.7 years and 38 years, respectively.

Overall, patients submitted to TVUS-guided fresh ET (Group 1) presented statistically higher pregnancy rates than the individuals submitted to TAUS-guided fresh ET (*p*=0.0107). The same was observed for the patients in the subgroups aged between 36 and 39 years (*p*=0.0037) and ≥40 years (*p*=0.0025). However, no significant differences between groups were observed in patients aged ≤ 35 years (*p*=0.0905), as seen in Table 1.

**Table 1:** Distribution of pregnancy for fresh embryo transfers (ET) with transvaginal (TVUS) and transabdominal (TAUS) ultrasound guidance.

	TVUS fresh ET		TAUS fresh ET		*p*
Age	Cycles	Pregnancy rates	Cycles	Pregnancy rates	
**≤ 35 years**	75	52.0%	61	44.0%	0.0905
**36 to 39 years**	63	47.6%	82	34.1%	0.0037*
**≥ 40 years**	83	28.9%	83	16.9%	0.0025*
**Total**	221	42.1%	226	30.5%	0.0107*

* Statistically significant; Z test.

## DISCUSSION

This retrospective study was conducted to present a modified TVUS technique used during fresh ET and to assess its efficacy in comparison with the conventional TAUS method in a group of unselected Brazilian women submitted to fresh ET. The findings demonstrated that patients submitted to fresh ET under TVUS guidance presented significantly higher pregnancy rates than patients submitted to TAUS-guided fresh ET, particularly in the group aged ≥ 36 years.

Improving implantation rates after embryo transfer has become an important quest in IVF; therefore, ultrasound guidance procedures deserve attention from the scientific community (Kojima *et al*., 2001). Because atraumatic ET is essential to IVF success (Schoolcraft, 2016), an important advantage of the TVUS-guided ET technique is the improved visualization of the details of the pelvic anatomy and the definition of the position of the transferred embryo (Kojima *et al*., 2001; Karavani *et al*., 2017). The catheter tip can be more clearly outlined than when under TAUS guidance, permitting optimal embryo deposition (Kojima *et al.,* 2001). This is an important aspect, since improved implantation rates have been described when embryos were discharged at a distance of 10 to 20 mm from the uterine fundus (Tiras *et al*., 2010).

TVUS-guided ET has been described as more effective than TAUS in women with retroverted uteruses and in obese patients, since it allows better and easier visualization (Paladini, 2009). In the present study, patients with retroverted uteruses and obese women undergoing TAUS-guided ET usually required a longer waiting time for bladder distention. Apart from the discomfort caused to patients by the requirement of having a full urinary bladder under TAUS and its associated problems (Fanchin *et al*., 1998; Lesny *et al*., 1998; Karavani *et al*., 2017), longer waiting times may also hinder the running of a busy clinic (Bodri *et al*., 2011). Moreover, uterus abnormalities such as endocervical crypts, tortuous cervical canals, marked uterine anteversion, and local causes such as isthmoceles, which may result in difficult transfers, can also be more easily analyzed and resolved under TVUS than TAUS guidance (Larue *et al*., 2017b). However, care must be taken concerning the correct positioning the TV transducer, which must be consistent with the anteverted or retroverted position of the uterus.

The main drawback attributed to TVUS-guided ET has been the difficulty in managing both the transducer and the catheter at the same time, which has been considered a challenging procedure when performed by a single operator (Hurley *et al*., 1991; Kojima *et al*., 2001). Previous papers have reported the assistance of either an embryologist, assigned the job of unloading the embryos (Bodri *et al*., 2011), or a nurse to hold the outer catheter while the physician manages the transducer and threads the inner catheter into position (Karavani *et al*., 2017). Because of the limited space available, these procedures tend to be awkward and uncomfortable for the physician, and possibly to the patient. In a study by Revelli *et al*. (2016), the authors suggested an alternative process to facilitate ET by previously measuring the uterine length with TVUS to calculate the optimal site for embryo discharge, to later conduct the ET either by clinical touch or under TAUS guidance. The authors claimed that a single operator might more easily conduct the procedure, but the results were similar to the ones obtained with TAUS guidance alone (Revelli *et al*., 2016).

When reading recent literature on ET, two important observations clearly emerge. First, the clinical outcomes of TVUS-guided ET procedures have been at least equal (Porat *et al*., 2010; Bodri *et al*., 2011; Cozzolino *et al*., 2018) or sometimes better (Larue *et al*., 2017a; Karavani *et al*., 2017) than conventional TAUS-guided ET. Secondly, improved techniques to allow TVUS to be executed by a single operator are being considered. In the present study, once the vaginal speculum was placed in the vagina, it was kept in position for the whole extent of the ET procedure. After ascertaining the uterine contour and degree of angulation with TVUS, the physician introduced the outer sheath of the catheter under the visualization. Once in the correct position, with the palm of his hand turned to himself, the physician held the transducer and the outer sheath between his thumb and index finger, therefore controlling both with just one hand. Then the physician introduced the inner catheter with his right hand, to then carefully drive it to the unloading area inside the uterus. While some dexterity is demanded to execute the TVUS technique, the learning curve should be short and result in less physical and emotional stress to physicians and patients, thus positively influencing the results.

The overall pregnancy rates obtained in the present study from TVUS- and TAUS-guided ET (42.1% *vs*. 30.5%) agreed with a large retrospective study (38% *vs*. 30%) conducted by Larue *at al*. (2017b). In both studies, pregnancy rates with TVUS guidance were significantly higher than with TAUS. Similarly, previous studies have also described significantly better clinical results with TVUS-guided ET (Kojima *et al*., 2001; Anderson *et al*., 2002). Interestingly, the outcomes of patients aged ≤36 years were not significantly different between the studied groups, although TVUS guidance produced better results than TAUS (52% *vs*. 44%).

The results presented herein must be interpreted in light of a series of limitations inherent to the study design. The groups contained women treated in two different moments, when only TVUS or only TAUS were performed in our service, which may have resulted in some selection and, possibly, performance bias. Nevertheless, all procedures followed the same in-house protocol and were performed by the same team, differing only the US procedure. Additionally, care was taken to select the patients that underwent fresh ET regardless of age, or any other physical or clinical characteristics. In another attempt to reduce selection bias and make the groups more homogeneous for comparison, only patients that underwent fresh ET were selected. As a result, the findings tied to the included population may be more broadly generalized for individuals undergoing IVF. Clinical pregnancy and live birth rates are more representative of overall IVF outcomes than chemical pregnancy rates. The decision to use β-hCG to calculate pregnancy rate as the main study endpoint was based on the fact that β-hCG tests were the first pregnancy assessment performed after ET and, in our view, they may more adequately reflect the efficacy of the US procedures. In later moments, other factors unrelated to ET may affect clinical pregnancy and life birth rates.

## CONCLUSION

Taking into consideration the limitations of this study and the observed pregnancy rates, the TVUS technique described for fresh ET was apparently at least as effective as TAUS in a group of unselected Brazilian patients. Pending further prospective studies to better ascertain the effect of TVUS during ET, the technique presented deserves consideration since it can offer better visualization, more comfort to patients, and requires only one operator, without negatively affecting pregnancy results.
